# Three-Layered Thin Films for Simultaneous Infrared Camouflage and Radiative Cooling

**DOI:** 10.3390/ma16114188

**Published:** 2023-06-05

**Authors:** Luyu Zhang, Wenjie Zhang, Yuanbin Liu, Linhua Liu

**Affiliations:** 1 School of Energy and Power Engineering, Shandong University, Jinan 250061, China; 2 Key Laboratory for Thermal Science and Power Engineering of Ministry of Education, Department of Engineering Mechanics, Tsinghua University, Beijing 100084, China

**Keywords:** multilayered thin films, infrared camouflage, radiative cooling, Fabry-Perot resonance cavity

## Abstract

With the rapid advancements in aerospace technology and infrared detection technology, there are increasing needs for materials with simultaneous infrared camouflage and radiative cooling capabilities. In this study, a three-layered Ge/Ag/Si thin film structure on a titanium alloy TC4 substrate (a widely used skin material for spacecraft) is designed and optimized to achieve such spectral compatibility by combining the transfer matrix method and the genetic algorithm. The structure exhibits a low average emissivity of 0.11 in the atmospheric windows of 3–5 μm and 8–14 μm for infrared camouflage and a high average emissivity of 0.69 in 5–8 μm for radiative cooling. Furthermore, the designed metasurface shows a high degree of robustness regarding the polarization and incidence angle of the incoming electromagnetic wave. The underlying mechanisms allowing for the spectral compatibility of the metasurface can be elucidated as follows: the top Ge layer selectively transmits electromagnetic waves ranging from 5–8 μm while it reflects those in the ranges of 3–5 μm and 8–14 μm. The transmitted electromagnetic waves from the Ge layer are first absorbed by the Ag layer and then localized in the Fabry-Perot resonance cavity formed by Ag layer, Si layer and TC4 substrate. Ag and TC4 make further intrinsic absorptions during the multiple reflections of the localized electromagnetic waves.

## 1. Introduction

With the rapid development of the aerospace industry, the infrared (IR) camouflage of spacecraft is attracting more and more research attention [1]. Full IR band (from near infrared to far infrared) low emissivity coatings have been commonly used in IR camouflage for years, although the IR radiation signals of spacecraft mainly depend on its temperature-dependent emissivity being within the mid-far IR atmospheric windows (3–5 μm and 8–14 μm) [2]. In this regard, the low emissivity of 5–8 μm significantly hinders the radiative cooling efficiency of a spacecraft, which may raise the temperature level and largely limit its camouflage performance. Hence, high performance IR camouflage coating demands favorable spectral selectivity, i.e., high emissivity in non-atmospheric window bands for radiative cooling [3] and low emissivity in the atmospheric window bands for IR camouflage [4].

Recently, multilayered thin films have emerged that efficiently tune the radiative properties of materials [5,6,7,8,9,10,11]. As a type of metamaterial, multilayered thin films are composed of two or more stack-arranged dielectric materials, which have great potential for applications in perfect absorbers (PAs) [5,11,12,13,14], radiative cooling [15,16,17,18,19] and IR camouflage [20,21,22,23,24,25,26,27,28]. While the investigation of individual functionalities is progressively advancing, the exploration of multilayered thin films that can be compatible with diverse functionalities is thriving. For example, Qi et al. [29] designed a one-dimensional photonic crystal structure based on ZnS/Ge to achieve visible and infrared compatible stealth. Deng et al. [30] proposed a low visibility and low thermal emission thin film structure for multispectral camouflage. Kang et al. [31] designed a plasma structure based on Ge_2_Sb_2_Te_5_ (GST) for tunable multi-band camouflage and radiative cooling. Zhao et al. [21] realized broad-spectrum infrared spectral selectivity through a 20-layered and a 23-layered thin film structure. Wang et al. [32] designed an IR camouflage-radiative cooling compatible structure consisting of 12 layers of thin film. Considering the structural simplicity for large-scale fabrication, Liang et al. [33] and Yang et al. [34] proposed a four-layered film structure to achieve the compatibility of IR camouflage and thermal management. Zhu et al. [35] and Pan et al. [36] achieved laser camouflage and visual camouflage alongside the compatibility of IR camouflage and radiative cooling by combining multilayered structures and surface microstructures, respectively. However, in most of the previous works, the structural parameters of the multilayered thin films were not rigorously optimized, while the polarization and incidence angle robustness of the structure were rarely included. Moreover, multilayered thin film structures with compatibility of IR camouflage and radiative cooling using fewer layers are highly desirable to further decrease the difficulties of large-scale fabrication. In addition, for a more comprehensive overview of the previously reported designs’ functionality and structure in various scenarios, we present a summary in Table 1.

This work proposes a three-layered Ge/Ag/Si thin film structure on a TC4 substrate for spectrally selective modulation to achieve compatibility with IR camouflage and radiative cooling. The material and thickness of each layer are optimized via a genetic algorithm (GA) [37,38,39,40], a powerful optimization method used for structural parameters that simulates the processes of inheritance and variation in biological populations. The final structure exhibits broad-spectrum low emissivity for IR camouflage in the atmospheric windows of 3–5 μm and 8–14 μm, and broad-spectrum high emissivity for radiative cooling in the non-atmospheric windows of 5–8 μm. Furthermore, the directional performance for different polarizations and the underlying mechanisms are discussed in detail. The results demonstrate that the compatibility can be maintained over a wide directional range of 0° to 80° and is insensitive to the polarization state.

## 2. Methodology

### 2.1. Design Process

**Figure 1:**
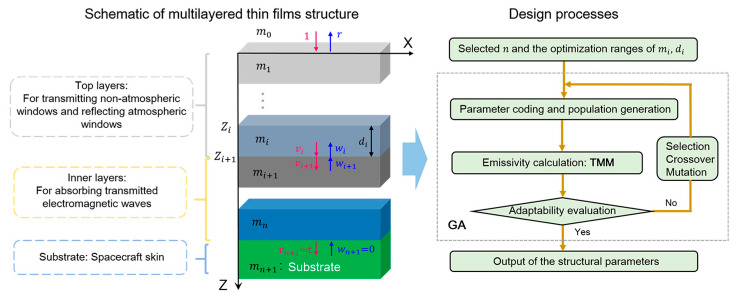
Schematic of the model and method for the design of multilayered thin films.

The schematic and design strategy of the multilayered thin films are illustrated in Figure 1. The structure is composed of *n* unit layers. The material and thickness of each layer are denoted as *m_i_* and *d_i_*, respectively. In principle, they are expected to achieve the spectral selectivity by combining the unique electromagnetic response of each stacked layer: (1) the top layers allow transmission in the non-atmospheric window band but reflect in the atmospheric window band; (2) the inner layers absorb the transmitted electromagnetic waves in the non-atmospheric window band. In the design process, the transfer matrix method (TMM) and the GA are combined to determine the optimized layer number *n*, the material *m_i_* and the thickness *d_i_* of each layer. Specifically, the GA is used for the structural parameters optimizing iterations, while the TMM is used to calculate the apparent emissivity in each iteration. The essentials of TMM and GA are presented below.

### 2.2. Emissivity Calculation for Multilayered Thin Films

The TMM is an accurate and efficient method used to calculate the reflectance and transmittance of multilayered thin films [41]. The TMM describes the forward and backward amplitude change in each film layer and at its boundaries via transfer matrix. For the structure shown in Figure 1, light with a normalized electric field amplitude of 1 passes through the *n* isotropic film layers from a vacuum (layer 0) to the substrate (layer *n* + 1). If we denote the forward and backward amplitude on the *i*th side by *v_i_* and *w_i_*, respectively, there are relationships between them [42]:

(1) $$v_{i+1}=\left(v_{i}e^{i\delta_{i}}\right)t_{i,i+1}+w_{i+1}r_{i+1,i}$$

(2) $$w_{i}e^{-i\delta_{i}}=w_{i+1}t_{i+1,i}+\left(v_{i}e^{i\delta_{i}}\right)r_{i,i+1}$$

where *r_a,b_* and *t_a,b_* denote the reflection and transmission coefficient from layer *a* to its adjacent layer *b*, respectively. *a* and *b* represent the layer number, like *i* or *i* + 1. *δ_i_* is the phase change passing through the *i*th layer, and is expressed as [42]:

(3) $$\delta_{i}=d_{i}\cdot k_{z,i}$$

*k_z_*_,*i*_ denotes the normal components of the complex wave vector in the *i*th layer [42]:

(4) $$k_{z,i}=\frac{2\pi }{\lambda }{\tilde{n}}_{i}\left(\lambda \right)\cos\left(\theta_{i}\right)$$

where ${\tilde{n}}_{i}$ and *θ_i_* represent the complex refractive index and the incident angle of the *i*th layer, respectively, while λ represents the wavelength.

Next, the amplitude change in each layer (*i =* 1, *…*, *n* − 1) can be expressed by the layer transfer matrix *M_i_* in the form [42]:

(5) $$\left(\begin{array}{l} v_{i}\\ w_{i} \end{array}\right)=M_{i}\left(\begin{array}{l} v_{i+1}\\ w_{i+1} \end{array}\right)=\left[\left(\begin{array}{cc} e^{-i\delta_{i}} & 0\\ 0 & e^{i\delta_{i}} \end{array}\right)\left(\begin{array}{cc} 1 & r_{i,i+1}\\ r_{i,i+1} & 1 \end{array}\right)\frac{1}{t_{i,i+1}}\right]\left(\begin{array}{l} v_{i+1}\\ w_{i+1} \end{array}\right)$$

The consequent amplitude change can be expressed by the total transfer matrix $\tilde{M}$ connecting each layer [42]:

(6) $$\left(\begin{array}{l} 1\\ r \end{array}\right)=\tilde{M}\left(\begin{array}{l} t\\ 0 \end{array}\right)$$

(7) $$\tilde{M}=\frac{1}{t_{0,1}}\left(\begin{array}{cc} 1 & r_{0,1}\\ r_{0,1} & 1 \end{array}\right)M_{1}M_{1}\cdots M_{n-1}$$

The reflectivity and transmissivity can be obtained by [42]:

(8) $$R={\left|r\right|}^{2}={\left|1/{\tilde{M}}_{11}\right|}^{2},T={\left|t\right|}^{2}={\left|{\tilde{M}}_{21}/{\tilde{M}}_{11}\right|}^{2}$$

When the reflectance *R* and the transmittance *T* are calculated, their corresponding absorbance *α* = 1 − *T* − *R* can also be calculated. In this study, the titanium alloy TC4 spacecraft skin is taken as the substrate, such that the transmittance of the structure is *t* = 0. Then the absorbance can be approximated as *α* = 1 − *R*. From Kirchhoff’s law, it is known that the absorbance is equal to the emissivity at thermal equilibrium, i.e., ε = α. In this study, we use the open-source program TMM to calculate the absorptivity and reflectivity of the multilayer film structure [43].

### 2.3. Genetic Algorithm

**Figure 2:**
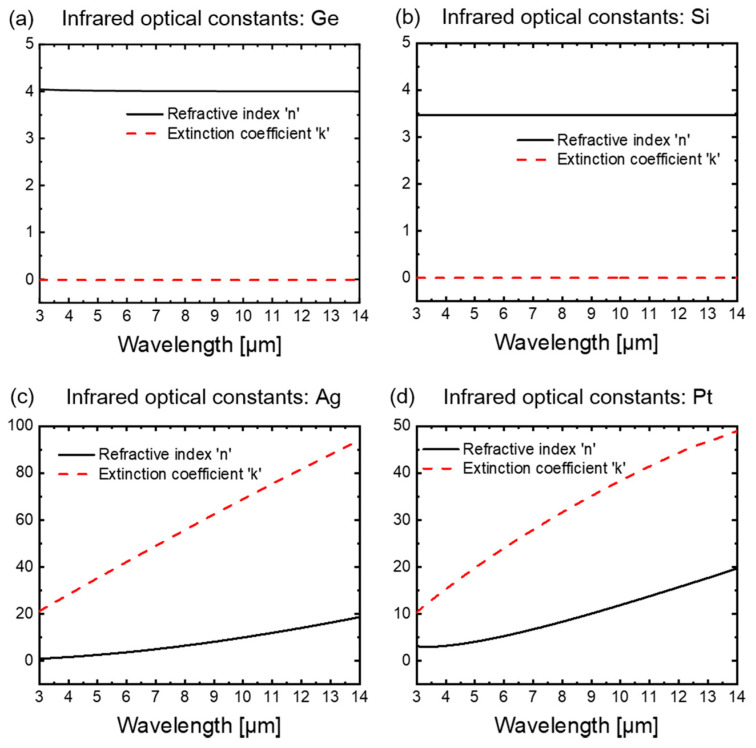
Infrared optical constants (*n*, *k*) of (**a**) Ge, (**b**) Si, (**c**) Ag, (**d**) Pt in the 3–14 μm range.

The GA is a biomimetic optimization method that simulates the phenomena of selection, crossover and mutation in biological genetics. The GA starts with a random initial population and iteratively generates individuals better suited to the environment through the manipulation of genetic operators. Eventually, the population evolves into an optimal solution within the defined parameter range [33,34]. Semi-conductor materials Ge and Si exhibit a stable high refractive index and near-zero extinction coefficients, while noble metal materials Ag and Pt exhibit a monotonically increasing high refractive index (*n*) and extinction coefficients (*k*) with wavelength in the 3–14 μm band. The infrared optical constants (*n*, *k*) for Ge [44], Si [45], Ag [46], and Pt [47] materials within the 3–14 μm band are depicted in Figure 2. The strategic combination of these materials, with specific thicknesses, has been demonstrated in the literature to possess superior selectively emissive characteristics [33,34]. Here, in order to realize the original design intention, i. e. achieving the spectral selectivity by combining different electromagnetic responses of the top layers, inner layers and the bottom layers, we take Ge, Si, Ag, and Pt as the candidate materials for the stacked structure, and encode the material of each layer as *m_i_*.

For practical engineering applications, the simplicity of large-scale fabrication is a critical factor, to which intensive attention should be paid. In order to achieve the original design intention with as few layers as possible, the number of top layers, inner layers, and bottom layers are all set to *n* = 1. Namely, the total layer number is *n* = 3. The candidate material of each layer is configured as: the first layer uses Ge or Si for the spectrally selective transmission of the incident light; the second layer uses the ultrathin metal layer Ag or Pt for absorption of the light transmitted from the first layer; the third layer uses Ge or Si for the resonance with the second thin metal layer to further enhance the absorption of the light transmitted from the second layer. The material of each layer will be determined throughout the GA optimization process. Alongside the candidate material *m_i_*, the thickness *d_i_* is the other key parameter affecting the apparent emissivity of the multilayered thin film structure. To avoid possible local optimum values, eight structures composed of different materials are evolved and screened with three genes per layer thickness *d_i_* as an individual.

**Figure 3:**
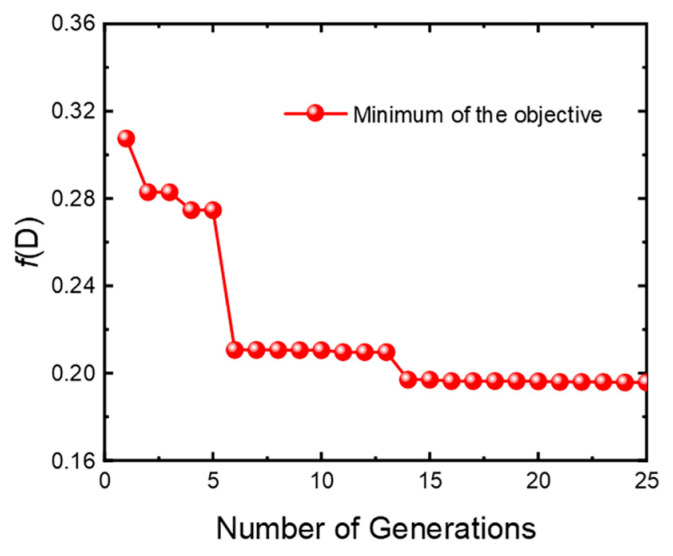
Evolution of the best objective value in the optimization for one set of layered films.

The optimization process begins by generating a population with different parameter combinations within the defined range, then the spectral emissivity levels in 3–14 μm of each individual combination are calculated by the TMM. Since IR detection and radiative cooling concerns integrated radiation power in certain spectral range, the adaptability of each individual with gene *D* = [*d*_1_,*d*_2_,*d*_3_] is evaluated through the objective function based on spectrally integrated average emissivity:

(9) $$f(D)=\sqrt{\frac{\sum_{j=1}^{3}g_{j}{\left[\varepsilon_{tar}(j)-\varepsilon_{cal}(j)\right]}^{2}}{3}}$$

where *ε_tar_* and *ε_cal_* represent the expected and calculated average emissivity, respectively. *j* = 1, 2, 3 denotes the three concerning bands, i.e., the mid-IR atmosphere window 3–5 μm, the radiative cooling band 5–8 μm, and the far-IR atmosphere window 8–14 μm, respectively. *g_j_* is the weight factor corresponding to the concerning bands, and the band with a relatively large weight factor will be especially focused on in the evaluation. Towards the IR camouflage and radiative cooling compatibility, the expected average emissivity in 3–5 μm, 5–8 μm and 8–14 μm are set to [0 1 0] and the weighting factor is set to [1 1 1], respectively. In each generation, good genes will be selected referring to low objective function values of the population, while the bad genes will be mutated. The selected good genes crossover and become the next generation of the initial population. The objective value continuously decreases with the iterative evolution process of the population. Convergence will be achieved when the relative difference of the objective value between two adjacent generations is smaller than 10^−4^ in this study:

(10) $$F=\left|f_{\beta +1}(D)-f_{\beta }(D)\right|/f_{\beta }(D)<10^{-4}$$

where *β* and *β* + 1 represent two adjacent generations. Eventually, the optimized results are obtained. In this study, the number of individuals in the random population is set to 40, and the maximum number of genetic generations is set to 25. The thickness range of each layer is set to 0.01–1 μm. Figure 3 illustrates the evolution of the objective function in the GA for one set of layered films. For all eight sets of structures, the GA achieves convergence within 25 generations.

## 3. Results and Discussion

**Figure 4:**
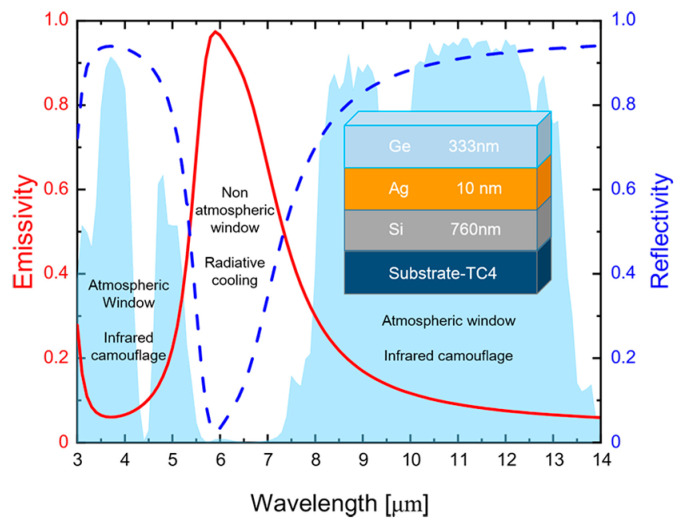
Spectral emissivity (red solid line) and reflectivity (blue dashed line) of the optimized multi-layered thin films. For convenience, the optimized structure is shown in the inset, in which the selected materials and thicknesses are also labeled. The shaded area represents the atmospheric transmittance.

The final optimized structural parameters are shown in Table 2. The structure includes a Ge film layer, an ultra-thin Ag film layer, and a Si film layer with a thickness of 333 nm, 10 nm, and 760 nm, respectively. The three layers are stacked on the TC4 titanium alloy substrate. In order to evaluate the radiative properties of the structure optimized by the GA, we calculated the normal spectral emissivity and reflectivity, as shown in Figure 4. The shaded area in the figure is the transmittance of the atmospheric window. It is shown that the optimized structure has broad-spectrum low emissivity in the 3–5 μm and 8–14 μm atmospheric window bands as well as broad-spectrum high emissivity in the 5–8 μm non-atmospheric window band. We further calculated the average emissivity of these target bands as ε_avg,3–5 μm_ = 0.11, ε_avg,8–14 μm_ = 0.11, ε_avg,5–8 μm_ = 0.69, respectively. The results demonstrate that the optimized structure has desirable infrared camouflage and radiative cooling compatibility.

**Figure 5:**
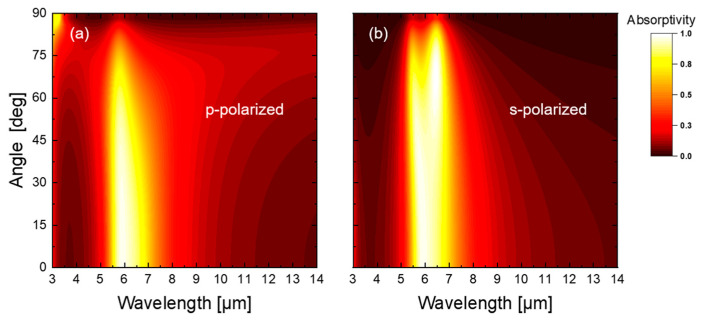
Polarized absorptivity of the optimized structure at different angles (**a**) *p*-polarized (**b**) *s*-polarized.

**Figure 6:**
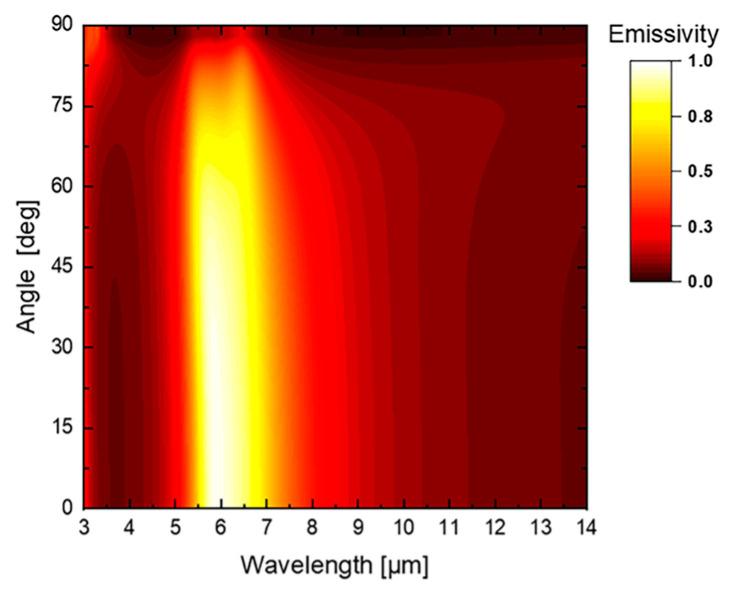
Emissivity of the optimized structure at different angles.

Emission direction and polarization characteristics are two more important factors in IR camouflage performance. Hence, we further examined the polarization and directional robustness of the optimized structure via the TMM. The spectral absorptivity of both *p*- and *s*- polarizations at different incidence angles are shown in Figure 5. It is shown that, for both *p*- and *s*- polarizations, the structure exhibits high absorptivity for non-atmospheric windows and low absorptivity for atmospheric windows over a wide angular range (0–80°). Since all of the thin films and the substrate are isotropic materials, according to Kirchhoff’s law, the emissivity of the optimized structure can be obtained by averaging the *s*- and *p*-polarized absorptivity, as shown in Figure 6. The results indicate that the infrared camouflage and radiative cooling compatibility of the coating is insensitive to direction and polarization.

**Figure 7:**
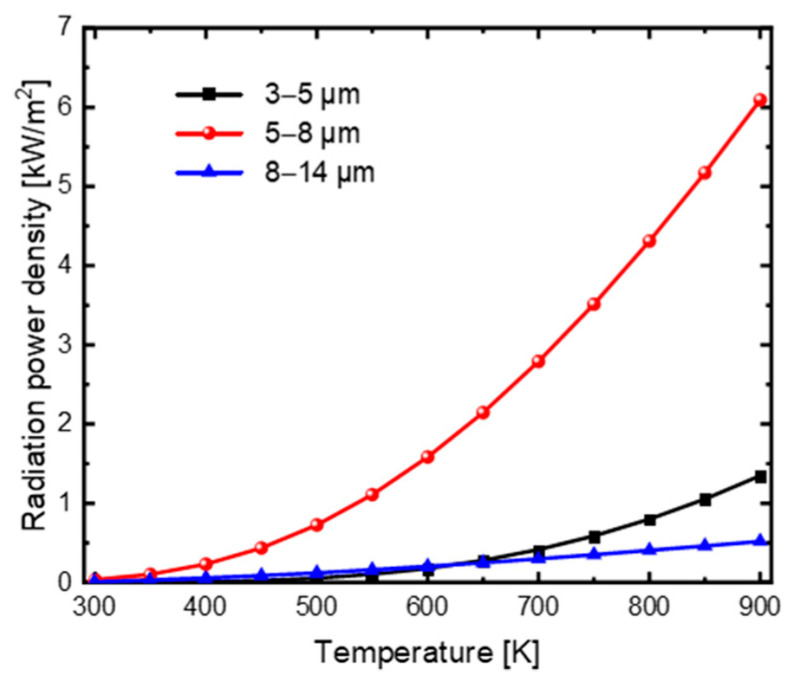
The emissive power density in 5–8 μm for radiative cooling, and the IR radiation signal power density in the detection bands of 3–5 μm and 8–14 μm.

In order to access the radiative cooling performance and camouflage performance of the three-layered film structure, we calculated the emissive power density in 5–8 μm for radiative cooling and the IR radiation signal power density in 3–5 μm and 8–14 μm for being detected by Equation (11) [31], respectively, where λ_1_ and λ_2_ are the minimum and maximum wavelengths of the integral spectra range, as shown in Figure 7. It can be observed that the radiative cooling power density increases significantly while the IR radiation signal power densities increase slightly with increasing temperature from 300 K to 900 K. Moreover, the remarkable increase of the radiative cooling power will contribute to the reduction of the spacecraft surface temperature, thus further suppressing the increase of the IR radiation signal intensity in the 3–5 μm and 8–14 μm detection bands. Consequently, the structure exhibits excellent compatible camouflage and radiative cooling performance, especially for high temperature spacecraft. In addition, the structure designed in this study achieves this compatibility with only three layers, which is simpler and more easily fabricated than those listed in Table 1.

(11) $$E_{\lambda_{1}-\lambda_{2}}(T)=\int_{\lambda_{1}}^{\lambda_{2}}\varepsilon (\lambda )E_{b}(\lambda ,T)d\lambda$$

**Figure 8:**
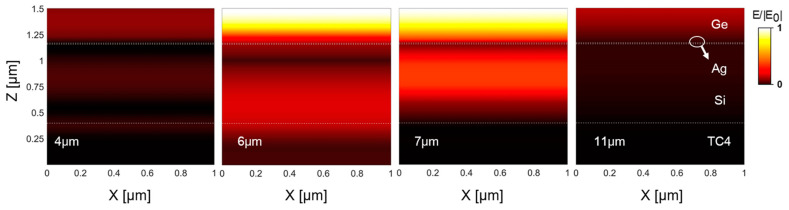
The internal normalized electric field distribution of the optimized structure at the wavelengths of 4 μm, 6 μm, 7 μm, and 11 μm. The white dash lines denote the interfaces between the layers, and the white circle indicates the ultrathin Ag layer.

**Figure 9:**
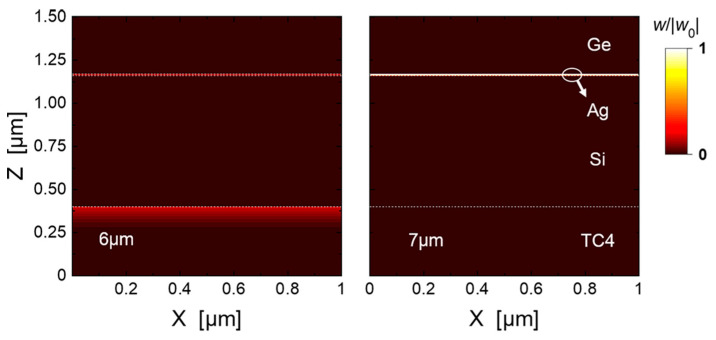
Normalized absorption distribution of the optimized structure at the wavelengths of 6 μm and 7 μm.

In order to clarify the mechanism of the spectrally selective emissivity, we examined the normalized electric field of the optimized structure at wavelengths of 4 μm and 11 μm in the atmospheric window and at wavelengths of 6 μm and 7 μm in the non-atmospheric window as examples, respectively, as shown in Figure 8. It can be seen that the electric field intensity of the structure at wavelengths of 4 μm and 11 μm are much lower than that at wavelengths of 6 μm and 7 μm, which indicates that the outer surface of the top Ge layer makes the first spectral selection. That is, the top Ge layer reflects off most of the electromagnetic waves in the atmospheric window bands (see 4 μm and 11 μm) but allows the electromagnetic waves in the non-atmospheric window band (see 6 μm and 7 μm) to penetrate into the interior of the stacked structure. Once the electromagnetic waves within the non-atmospheric window enter the interior of the structure, they are first attenuated during their passage through the top Ge layer, and subsequently the remaining waves propagate onto the ultra-thin Ag layer. The Ag layer plays a key role in two aspects. One aspect is that the thin Ag layer forms the Fabry-Perot (F-P) resonance [48] together with the Si layer and the titanium alloy substrate. The F-P resonance localizes the electromagnetic waves in the cavity. The other aspect is that the thin Ag layer can efficiently absorb both the localized electromagnetic waves and the transmitted electromagnetic waves from the Ge layer. Figure 9 illustrates the absorption distribution of the optimized structure at 6 μm and 7 μm under normal incidence, where the power absorption density (W/m^3^) is defined as [49,50]:

(12) $$w\left(x,y,z\right)=\frac{1}{2}\varepsilon_{0}\omega \varepsilon^{\prime \prime }\left(x,y,z\right){\left|E\left(x,y,z\right)\right|}^{2}$$

where *ε*_0_ and *ε*^″^ denote the permittivity of vacuum and the imaginary part of the dielectric function of the stacked layers, respectively. *ω* represents the angular frequency, and ***E*** is the electric field vector. It is shown that, alongside the Ag layer, the titanium alloy substrate can further absorb the localized waves in the F-P resonance cavity.

**Figure 10:**
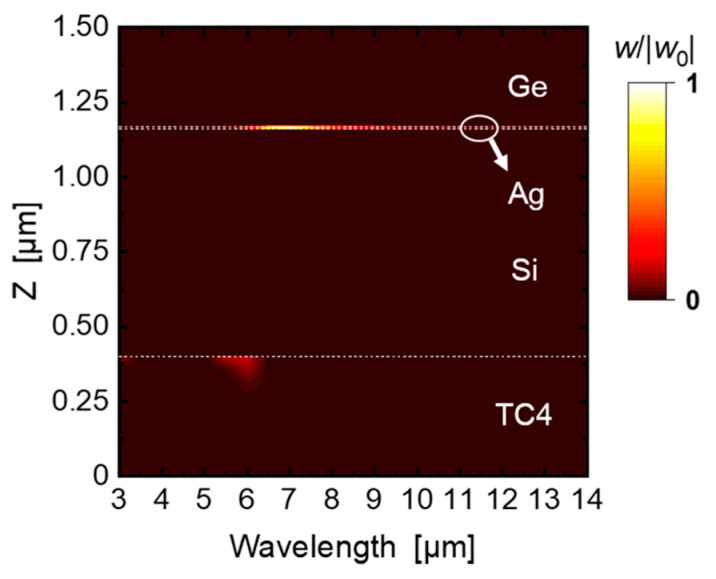
Normalized spectral absorption distribution of the optimized structure.

Figure 10 illustrates the spectral absorption distribution in the entire 3–14 μm band. It can be seen that the greatly enhanced absorption mainly focuses on the thin Ag layer in 6–8 μm, and a weaker absorption occurs in the TC4 substrate in 5–6 μm. The combination of the absorption by the Ag layer and the TC4 substrate leads to the apparent high emissivity of the optimized structure in the 5–8 μm non-atmosphere window for radiative cooling. Although the top Ge layer also absorbs within this band, the power absorption density is much lower than the Ag layer and the substrate. In addition, the absorption density in the 3–5 μm and 8–14 μm ranges is much lower than that in the 5–8 μm range, which is the reason for the low emissivity in the atmospheric window bands. Consequently, it is verified that the optimized structure realizes the IR camouflage and radiative cooling compatibility with the electromagnetic response combination of each layer. In this study, we used the open-source software S4 (version 1.1.1) [51] to calculate the internal normalized electric field distribution and normalized absorption distribution in the optimized structure.

**Figure 11:**
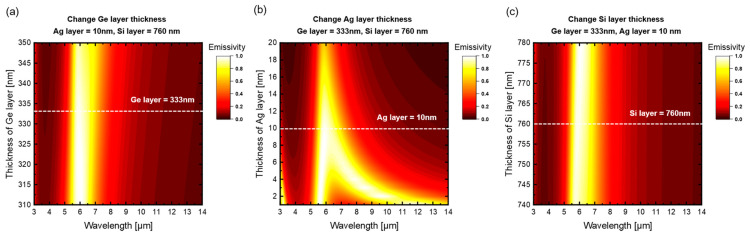
Sensitivity of the spectral emissivity to the thickness of each layer: (**a**) Ge layer, (**b**) Ag layer, (**c**) Si layer. The white dashed lines represent the desired thicknesses optimized by GA.

Considering the uncertainties in the fabrication process, the film thickness of each layer of the samples or products will deviate from the optimized value, so that the apparent spectral emissivity of the structure will be influenced. In order to quantitatively evaluate the thickness sensitivity, we calculate the apparent spectral emissivity of the three-layered film structure for varying thickness of each layer with the single variable principle, respectively. The results are illustrated in Figure 11, where the white dashed lines represent the desired thicknesses optimized by the GA. It can be seen from Figure 11 that the apparent spectral emissivity remains nearly unchanged within a Ge/Si layer thickness variation range of approximately ±20nm while it changes remarkably within the Ag layer thickness variation range of only approximately ±10nm. The sensitivity difference between Ge/Si layer thickness and Ag layer thickness can be attributed to the different roles of each layer. Since the Ag layer simultaneously plays the roles of both forming the F-P resonance cavity and absorbing the transmitted electromagnetic waves from the Ge layer and localized electromagnetic waves in the F-P resonance cavity, its thickness becomes the key factor affecting the apparent spectral emissivity of the structure. Hence, the thickness of Ag layer should be specially and accurately controlled in the manufacturing process.

## 4. Conclusions

In order to achieve compatible infrared camouflage and radiative cooling for spacecraft, a three-layered Ge/Ag/Si thin film structure on a TC4 substrate is designed via a GA and a TMM. The optimized structure can realize broad-spectrum low emissivity of 0.11 in the 3–5 μm and 8–14 μm atmospheric window bands for IR camouflage, and high average emissivity of over 0.69 in the 5–8 μm non-atmospheric window bands for radiative cooling. By calculating the radiative cooling power density and the IR radiation signal power density, we demonstrate the excellent camouflage and cooling compatibilities of the optimized structure, especially for high temperature spacecraft. Moreover, this compatibility can be maintained over a wide directional range of 0–80° for different polarizations. The mechanism analysis demonstrates that the compatibility can be attributed to the spectral selectivity of the top Ge layer, the intrinsic absorption of the inner Ag layer, and the excitation of the Fabry-Perot resonance formed by Ag, Si and TC4 substrate. The optimized structure achieves the compatibility of radiative cooling and IR camouflage with only three layer films, which is expected to be more applicable for large-scale fabrication and practical engineering applications.

## Figures and Tables

**Table 1:** Comparison of representative theoretical and experimental works on thermal camouflage compatibility in recent years.

Work by	Multilayered Films Only	Number of Layers	3–5 μm Infrared Camouflage	5–8 μm Radiative Cooling	8–14 μm Infrared Camouflage
Qi et al. [29]	√	8	√	×	×
Deng et al. [30]	√	6	√	×	√
Kang et al. [31]	×	×	√	√	√
Zhao et al. [21]	√	20/23	×	√	√
Wang et al. [32]	√	12	√	√	√
Liang [33] and Yang [34] et al.	√	4	√	√	√
Zhu [35] and Pan [36] et al.	×	×	√	√	√

“√” and “×” denote “yes” and “no”, respectively.

**Table 2:** The optimized parameters of the compatible multilayered thin films structure.

	Layer 1	Layer 2	Layer 3	Substrate
Material	Ge	Ag	Si	TC4
Thickness/[nm]	333	10	760	/

## Data Availability

The data that support the findings of this study are available from the corresponding author upon reasonable request.
